# Symptomatic Venous Thromboembolic Events in COVID-19 Patients after Hospital Discharge: Aspects to Consider

**DOI:** 10.31083/j.rcm2306219

**Published:** 2022-06-20

**Authors:** Călin Pop, Anca Hermenean, Liana Moș, Coralia Cotoraci

**Affiliations:** ^1^Department of Biology and Health Sciences, Faculty of Medicine Arad, “Vasile Goldis” West University, 310048 Arad CP, Romania

**Keywords:** venous thromboembolism, COVID-19, anticoagulation, post discharge thromboprophylaxis

## Abstract

Venous thromboembolic (VTE) events have been increasingly reported in patients with coronavirus disease 2019 (COVID-19) after hospital discharge. Acute pulmonary embolism (PE) is the most frequent type of post-discharge VTE complication. Levels of procoagulants (fibrinogen, factor VIII, von Willebrand factor), and D-dimer are higher during the SARS-CoV-2 infection. Patients with more severe inflammatory and procoagulant response experience higher VTE rates during hospitalization, while the risk after hospital discharge have not been well characterized. The incidence of VTE events following hospitalization is heterogeneous, ranging from low (3.1 per 1000 discharges), to 1.8%, which appears higher than for other medical condition. This discrepancy was partially explained by the differences in VTE screening and follow-up strategies, and by the period when the information about the VTE was collected. These data were based mainly on observational and retrospective studies; however, evolving data are to come after the completion of the prospective trials. The current guidelines do not recommend routine post-hospital VTE prophylaxis for COVID-19 patients but recommend it for all hospitalized adults. A careful risk-benefit assessment of VTE probability should be performed, to determine whether an individual patient may merit post-discharge thromboprophylaxis. A score such IMPROVE DD can help identify the patient who will potentially benefit but is also important to consider the bleeding risk and the feasibility. The optimal duration and the type of extended thromboprophylaxis is still under debate (from a minimum of 14 days to a maximum of 42 days), and future studies will help to validate these protocols in different populations. Direct oral anticoagulants (DOACs), warfarin and low molecular weight heparin (LMWH) are recommended, but low doses of DOACs rather than LMVH or warfarin were predominantly used in most patients. Finally, the COVID-19 patients should be educated to recognize and advised to seek urgent medical care should VTE events occur after hospital discharge.

## 1. Background

### Case Scenario

A male patient with mild COVID-19, hospitalized (diagnosis was made via SARS-CoV-2 PCR in a certified laboratory) and discharged from a dedicated hospital, had to be readmitted 21 days later, because of new onset of symptoms, including intense dyspnea, cough, and cyanosis, after initial resolution of symptoms. An Angio CT scan revealed bilateral distal pulmonary segmental thrombi and elevated D-Dimer, suggesting acute PE. The ECG showed sinus tachycardia (110–120 bpm) and right axis deviation. A lower extremity venous duplex ultrasound was performed, without deep vein thrombosis (DVT) identification. The patient was overweight, having a body mass index (BMI) of 29. During the initial hospitalization he had received prophylactic anticoagulation (Enoxaparine 0.6 ml s.c. once daily) for 7 days and his SARS-CoV-2 nasopharyngeal swabs had been negative on the day of the initial discharge as well on the admission for PE. His inflammation markers were only slightly elevated. The patient was start on LWMH and then NOAC anticoagulation therapy and recovered well.

A high incidence of VTE events was reported in hospitalized patients, often despite thromboprophylaxis, during the COVID-19 pandemic [1, 2]. The most frequent thrombotic complication was PE, as part of VTE [3, 4]. As a result, anticoagulation strategies, with LMWH delivered at intermediate or therapeutic doses, has been established to improve the outcome for such patients. Currently, guidelines as well as experts’ opinions recommend the use of standard VTE thromboprophylaxis in all hospitalized patients who do not have suspected or confirmed VTE [5, 6, 7]. Nevertheless, many reports and investigations showed that a variable risk of COVID-19-associated VTE extends over the first 3 months after hospital discharge, but the cumulative incidence of such events has not been clearly determined [8, 9, 10, 11, 12, 13, 14, 15, 16, 17, 18, 19, 20, 21, 22, 23, 24, 25, 26, 27, 28, 29, 30, 31, 32, 33, 34, 35]. In this context, the American Society of Hematology (ASH) updated 2021 guidelines do not recommend extended anticoagulation in the COVID-19 patients discharged from the hospital, who do not have suspected or confirmed VTE, or another indication for anticoagulation [36]. However, this recommendation has a low level of certainty, in the absence of strong evidence, based on randomized controlled trials (RCT) to assess the true VTE incidence and the role of thromboprophylaxis for such patients. The purpose of this short review is to discuss the many branching decision points and options for prophylactic post-discharge anticoagulant treatment in COVID-19 patients.

## 2. The Pathogenesis of VTE Events in COVID-19 Patients

Thrombosis is as an important complication in acute COVID-19 hospitalized patients, with VTE occurring in between 8% and 23% of such patients [1]. SARS-CoV-2 infection produces an inflammatory and immunologic storm with a hyper inflammatory state, cytokines release, diffuse endothelial vascular damage, and fibrinogen consumption coagulopathy. Usually, high levels of C reactive protein (CRP), lactate dehydrogenase (LDH), ferritin, interleukins, fibrinogen, factor VIII, von Willebrand factor and D-dimer are present [37]. Furthermore, these responses predispose to widespread thrombotic vascular lesions with microangiopathy, disrupted cell membranes, and new vessel growth [38]. The patients with marked elevation of D-dimer and fibrin degraded products had the worst prognosis and a higher severity of COVID-19 illness [39].

The mainly localization of thrombus for those with PE was basal (segmental or sub segmental), where the pulmonary inflammation is probably most diffuse. Concomitantly, the absence of signs of DVT in venous duplex ultrasound suggests pulmonary thrombosis rather than embolism. The question regarding pulmonary thrombosis, embolism, or a combination thereof, may remain unaddressed, because of the limited number of the available autopsy studies [40, 41, 42, 43, 44, 45, 46]. However, the differences in the immunologic response could contribute to the different VTE/PE scenarios seen in the current studies. This pathway triggered by the viral infection overlaps with the classical pathway in the presence of different VTE provoking factors such as bed rest, failure to mobilization after discharge, hypoxemia, the presence of catheters, age, cancer, and other concomitant medical and nonmedical conditions. It is also plausible that the classical pathway become predominant after the resolution of infection, but for many of the patients with post-discharge VTE an important feature was the absence of the pre-existing VTE risk factors, which suggests that COVID-19 disease itself may be a risk factor.

The COVID-19 pathophysiology characterized by release of inflammatory cytokines such as IL-1 and IL-6 could explained this risk as a true immunothrombosis phenomenon. These cytokines inhibit fibrinolysis and natural anticoagulants and promote thrombosis by activation of endothelium, monocytes, platelets and the tissue factor VIIa pathway [47]. However, the treatment with IL-6 antagonist failed in demonstrating a lower risk of VTE events in COVID-19 patients, but a potential confounding factor appears when administered simultaneously with standard prophylactic anticoagulation [48].

A double peak evolution of D-dimer was frequently seen in patients with VTE after discharge, with an initial and late marked elevation of the D-dimer levels, equating to a 5 to 200-fold increase above the upper limit of normal [8, 9, 10, 11, 12, 13, 14, 15, 16, 17, 18, 19, 20, 21, 22, 23]. One prospective study in those patients followed the evolution of D-dimer and CRP and showed that 36% had D-dimer values above the cut-off of 500 ng/mL at outpatient follow-up, while their CRP levels were low. Despite the elevated D-dimer, the incidence of VTE was low for these patients [32]. Therefore, the importance of D-dimer or inflammations markers such as IL-6 as predictors for post-discharge VTE events in Covid-19 patients remain to be investigated. Finally, the risk of thrombosis after acute COVID-19 disease seems most probably related to the inflammatory and immunologic storm, although how long this persists is unknown.

## 3. Incidence and Risk of VTE after Discharge

The many published case reports induced the belief that COVID-19 patients had a higher incidence of post-discharge VTE events than other acute ill patients [8, 9, 10, 11, 12, 13, 14, 15, 16, 17, 18, 19, 20, 21, 22, 23]. The available data coming from heterogeneous sources (small and medium sample size predominantly retrospective studies, one large retrospective study, few prospective studies, 1 registry and 1 meta-analysis), generally with greater variability in their systematic follow-up and outcomes (mostly without systematic VTE screening), suggest a relatively low incidence of post discharge VTE, less than 2–3% [24, 25, 27, 28, 29, 31, 32, 49, 50, 51, 52, 53, 54].

The variable reported incidence across the studies could be explained by the different lengths and methods of follow-up, and by the different policies of post discharge anticoagulation: e.g., 0.2% in a multi center study of 1529 patients, 0.7 % in 485 consecutive patients with systematic screening for VTE 6 weeks after discharge, 1.1% in a rehabilitation cohort of 454 patients, 1.55% in a registry of 4906 patients, 2.5% in a single-center report of 163 patients, 2.6% in 152 patients discharged from the hospital without an indication for anticoagulation [24, 25, 29, 31, 32, 35]. Even, in those with severe forms of acute COVID-19 (390 participants from a Chinese study) no post discharge DVT was found at ultrasonography study of lower extremities [47]. Only one study systematically screened all discharged patients for both DVT and PE, and only one asymptomatic DVT (0.7%) and one symptomatic PE (0.7%) were diagnosed [32]. However, the asymptomatic or atypical presentations could remain undiagnosed and the data have been underestimated the incidence of post discharge VTE, as we know that many thromboembolic events are asymptomatic in critically ill patients [55]. In one prospective study the COVID-19 patients who had displayed at least one of the symptoms suggestive for VTE, but did not present for medical evaluation, were invited for a medical check-up and, if deemed necessary assessed with specific imaging. A total of 228 patients reported potential symptoms at telephone contact, but only one VTE event (acute PE) was diagnosed [31]. In another study, none of the post discharge VTE events were asymptomatic in the COVID-19 cohort. The autors performed specific analysis to estimate the effect of having missed 10% to 50% of asymptomatic VTE events and found that the absolute incidence of post discharge VTE remains at 0.8% (low), even if 50% of cases had been missed [28]. These data suggest that no deviations from standard work-up should be made for VTE diagnosis and treatment in COVID-19 patients during hospitalisation or after discharge.

A recent meta-analysis of 11 studies reporting the incidence of VTE (symptomatic and asymptomatic) after discharge (maximum at 180 days) in 18949 COVID-19 patients showed that the cumulative incidence of VTE events ranged between 0.2 and 14.8%, with a pooled incidence of 1.8% (95% CI (Confidence Interval): 0.8–4.1%, $I^{2}$ = 96.0%). A supplementary sub analysis of the studies enrolling more than one-hundred patients showed a VTE incidence of 1.63% (95% CI: 0.4–2.0%, *p <*0.0001, $I^{2}$: 96.0%). The incidence of acute PE ranged between 0.2 and 5.6%, with a pooled cumulative incidence of 1.5% (95% CI: 0.5–4.0%, $I^{2}$ = 93.4%) and represents the most frequent type of VTE complication. The incidence of DVT ranged between 0.1 and 2.6%, with a pooled cumulative incidence of 0.9% (95% CI: 0.3–2.1%, $I^{2}$ = 78.4%) [56].

The reported data regarding the administration of tromboprophylaxis or therapeutic anticoagulation after discharge are reported by few studies.The patients discharged without thromboprophylaxis had low incidence of VTE, comparable to the risk of post-discharge VTE in the generally medical patients [24, 25, 27, 28, 29, 31, 32, 46, 51, 54]. Generally, the incidence of VTE events in medical patients is higher in the first 3 weeks and lowers after 6 weeks following the discharge from hospital, and the rates of symptomatic VTE events range from 1% to 4% [57, 58]. The incidence of post discharge VTE events in COVID-19 patients was reported at 30 days on most of the studies and there is no evidence that patients with COVID-19 had a different pattern of incidence risk than other medical patients. Finally, we must wait the results of ongoing or prematurely stopped studies such as CORE-19, CISCO-19, NCT04508439, COVID-PREVENT (NCT04416048), PREVENT-HD (NCT04508023), to have more data about the incidence and risk rates of late VTE complications [35, 59, 60, 61, 62]

## 4. Risk Factors of Post Discharge VTE

The rate of VTE events was 2.0% within 30 days after discharge in a recent retrospective study of 447 patients hospitalized for COVID-19. No risk factor was associated significanly with the risk for these events [51]. However, the patients with a history of prior VTE (OR 3.24; 95% CI: 1.34–7.86%), D-dimer level greater than 3 μg/mL (OR, 3.76; 95% CI: 1.86–7.57%), and predischarge CRP level greater than 10 mg/dL (OR, 3.02; 95% CI: 1.45–6.29%) were predisposed to post discharge VTE (rate 1.3%) in another much larger retrospective study with 2832 patients [53]. At opposite ends, the CRP levels were low in a prospective study of 485 consecutive patients with COVID-19, and despite the highly elevated D-dimer in 36% of them, the incidence of post discharge VTE was low [32]. It is possible and remains to be investigated if the elevated D-dimer levels post-COVID-19 are markers of lung damage sequelae. Also, others clinical risk factors have been found to be associated with VTE after COVID-19, such as advanced age, male sex, intensive care unit hospitalization, cardiovascular and chronic kidney disease [1, 35]. The meta-regression analysis of the only published meta-analysis revealed significantly associations only with age, male gender and an inversely correlation with the length of follow-up. No associations were found for post discharge thromboprophylaxis, VTE history, cancer, intensive care stay and mean length of hospitalization [56]. The identification of such risk markers could help to build a score, to determine whether an individual COVID-19 patient may has extended thromboprophylaxis.

Until now, no such score is available, but the modified International Medical Prevention Registry on Venous Thromboembolism (IMPROVE VTE) and elevated D-dimer level score — IMPROVE DD (adding D-dimer values if × 2 upper limit of normal to IMPROVE VTE score), have been empirically used to select the high-risk COVID-19 patients with an increased risk for VTE [63, 64, 65]. In the CORE-19 registry, an IMPROVE VTE score of 4 or higher was associated with an elevated risk of late VTE, while in another study it was not associated with post discharge VTE [35, 53]. A possible explanation is that the identified risk factors to build the IMPROVE VTE score in general hospitalized patients (age >60 years, thrombophilia, immobilized ≥7 days immediately prior to and during hospital admission, active cancer, intensive care hospitalization, lower-limb paralysis) were not fully involved in the pathogenesis of thrombosis in the COVID-19 patients.

A retrospective study on 394 COVID-19 patients proposes a D-dimer cut-off of 2500 ng/mL (normal range <400 ng/mL) for the PE diagnostic and showed that D-dimer values are associated with an increased risk of death (if >1000 ng/mL) and have an important prognostic role [66]. The presence of residual elevated D-dimer in different studies suggests a possible role of ongoing coagulation and fibrinolysis, while other studies suggest a role for pulmonary microvascular thrombi in the pathogenesis of long Covid syndrome, with a reported reduced lung diffusing capacity up to 6 months post COVID-19 [65, 66, 67, 68]. Therefore, it seems that adding laboratory values such D-dimer level is of interest to identify also the patients who merit extended anticoagulation after discharge, while it predictive role remains unknown and larger prospective studies are required in order to validate the value of high D-dimer levels in these circumstances [32, 37, 39, 69]. Therefore, thromboprophylaxis or an anticoagulation decision in COVID-19 patients only on laboratory parameters (D-dimer, PT, aPTT, platelets, and fibrinogen) are recommended for hospitalized COVID-19 patients, but seems redundant after discharge without considering the patient’s complete clinical status or the specific imagistic results [7, 70].

## 5. Should COVID-19 Patients Receive Prophylactic Anticoagulation after Hospital Discharge?

Until now, two RCTs have reported results for post discharge anticoagulation after COVID-19. The ACTION trial (Therapeutic versus prophylactic anticoagulation for patients admitted to hospital with COVID-19 and elevated D-dimer concentration) showed no benefits for rivaroxaban 20 mg daily during hospital stay and continued after discharge for 30 days, compared with prophylactic LMWH administered only in hospital. However, the real efficacy of post discharge thromboprophylaxis cannot be evaluated in this study because the patients and the outcomes were only reported at 30 days after hospitalization [71].

The MICHELLE trial (Rivaroxaban versus no anticoagulation for post-discharge thromboprophylaxis after hospitalization for COVID-19: an open-label, multicenter, randomized, controlled trial), published on December 2021, compared treatment with rivaroxaban 10 mg daily versus placebo, in a population of 320 selected patients with increased VTE risk based on the IMPROVEDD and IMPROVE VTE scores (≥4 independent of the D-dimer level at discharge, or 2–3 with a D-dimer >500 ng/mL). The primary composite endpoint was the rate of symptomatic or fatal VTE events, asymptomatic DVT at venous duplex ultrasound or PE at Angio pulmonary CT, symptomatic arterial thromboembolism, and cardiovascular death at day 35. The results showed in the active group a significant reduction of thrombotic events and death after 35 days of treatment with a relative risk reduction of 33%, 95% CI: 0.12–0.90%; *p* = 0.02. Rivaroxaban was administered only after standard parenteral thromboprophylaxis during hospitalization and no increase in bleeding events was reported during follow-up at day 35 [72]. These results provide quite good level of evidence (the open-label design from MICHELLE trial has a potential risk of bias) about the role of extended thromboprophylaxis for VTE events in COVID-19 patients and high risk, such as those with an IMPROVE VTE score 2–3 plus increased D-dimer levels or an IMPROVE VTE score of 4 or more.

Currently published guidelines advise no routine anticoagulation for the post discharge patients who do not have suspected or confirmed VTE or another indication for anticoagulation [36, 64, 73, 74, 75]. However, the low certainty of the evidence represents an important limitation of these guidelines. Therefore, the guidelines recommend an individual assessment of the VTE and bleeding risks, to identify those patients who could benefit from ongoing prophylactic anticoagulation. The International Society on Thrombosis and Hemostasis (ISTH) guideline suggests such evaluation for all hospitalized COVID-19 patients, using clinical features (e.g., advanced age, past VTE, persistent reduced mobility, comorbidities like thrombophilia, obesity and intensive care stay), or a score like IMPROVE-DD [73]. An online calculator is available to estimate the 3-month risk of VTE based on four risk factors (https://www.outcomes-umassmed.org/improve/, accessed: 3 February 2022) and a separate calculator estimates the 3-month risk of VTE and also the bleeding risk, based on seven to eleven factors which were present prior to and during hospitalization (www.outcomes-umassmed.org/IMPROVE/risk_score/index.html). The COVID-19 patients should have medical education before discharge, to identify the signs and symptoms of VTE and should be advised to obtain a medical opinion without delay if these develop [76].

## 6. Duration and Type of Anticoagulation 

It is not possible with the actual level of evidence and data to make specific recommendations about the type and duration of extended prophylactic anticoagulation after COVID-19. The latest ASH guideline update from July 2021 on post-discharge thromboprophylaxis, considers based on indirect evidence and with a very low level of certainty that post-discharge thromboprophylaxis may reduce the risk of PE (OR: 0.76, 95% CI: 0.46–1.25%), VTE (OR: 0.76, 95% CI: 0.46–1.25%) and the risk of mortality (OR: 0.55, 95% CI: 0.37–0.83%), while it may increase the risk of major bleeding (OR: 1.52, 95% CI: 0.86–2.67%) [36]. The ISTH guideline recommends both LMWH and a DOAC to be used for extended thromboprophylaxis. The duration of post discharge treatment can be approximately 14 days at least and up to 30 days (evidence level 4) [75]. The Global COVID-19 Thrombosis Collaborative Group recommends LWMH or DOACs for up to 45 days for those patients with high risk of VTE, while CHEST guidelines recommend 35 to 42 days after hospital discharge [73, 74]. The Scottish Intercollegiate Guidelines Network (SIGN) guidelines also recommend LVMH or DOACs for up to 14 days, emphasizing that the choice and duration of extended thromboprophylaxis will depend on clinical judgment [76].

The regimen of extended LVMH thromboprophylaxis is not established and may follow the ASH guideline, which suggests a prophylactic-intensity to be use in critically ill patients with COVID-19 who do not have suspected or confirmed VTE [36]. There are some concerns about the use of DOACs in COVID-19 patients, due to the possibility of an increased bleeding risk and the potential for organ dysfunction, when administered with some antiviral treatments or multiple medications used for COVID-19 treatment [75, 77]. It is important to note that until now none of the DOACs accepted for use has a license for thromboprophylaxis in medical or COVID-19 patients after hospital discharge. Therefore, patient consent needs to be obtained and local policies should be followed. However, we have some data about DOACs, coming from published or ongoing studies: (1) rivaroxaban 20 mg/day used during hospital stay and 30 days after discharge increased bleeding compared with prophylactic anticoagulation (ACTION trial), while in another study the 10 mg dose/daily extended for 35 days to the high risk VTE patients improves clinical outcomes (MICHELLE trial); (2) the APOLLO trial (NCT04746339) is comparing if apixaban 2.5 mg twice per day versus placebo reduce mortality after hospital stay—Table 1 (Ref. [71, 72, 78]). All these trials test low doses of DOACs with the aim to minimize the bleeding risk as the use of extended prophylactic anticoagulation should consider the individual balance between VTE and bleeding risks.

**Table 1.** **Completed and ongoing trials of anticoagulants after COVID-19 infection**.

Trials	Status	Intervention	Results
1. Therapeutic versus prophylactic anticoagulation for patients admitted to hospital with COVID-19 and elevated D-dimer concentration (ACTION): an open-label, multicentre, randomized, controlled trial [71]	Published:	Rivaroxaban 20 mg daily during hospital stay and continued after discharge for 30 days, compared with prophylactic LMWH administered only in hospital	No benefits
	Lancet. 2021 Jun 12; 397 (10291): 2253–2263		
2. Rivaroxaban versus no anticoagulation for post-discharge thromboprophylaxis after hospitalization for COVID-19 (MICHELLE): an open-label, multicentre, randomized, controlled trial [72]	Published:	Rivaroxaban 10 mg daily versus placebo for 35 days after discharge	Reduction of thrombotic events and death after 35 days of treatment with a RRR of 33%, 95% CI: 0.12–0.90%; *p* = 0.02
	Lancet. 2022; 399 (10319): 50–59		
3. Apixaban for Prophylaxis of thromboembolic Outcomes in COVID-19: the Apollo Trial [78]	Ongoing:	Apixaban 2.5 mg twice daily versus placebo for 30 days after discharge	Recruiting
	ClinicalTrials.gov Identifier: NCT04746339		
4. Effect of the Use of Anticoagulant Therapy During Hospitalization and Discharge in Patients With COVID-19 Infection [60]	Ongoing:	This trial will evaluate prophylactic and full-dose enoxaparin administered in hospital followed by rivaroxaban 10 mg/day for 30 days in comparison to no intervention	Recruiting
	ClinicalTrials.gov Identifier: NCT04508439		Estimated enrollment 130 patients
5. XACT – Trial: Factor Xa Inhibitor Versus Standard of Care Heparin in Hospitalized Patients With COVID-19 [79]	Ongoing:	150 patients were randomized 1:1 to subcutaneous enoxaparin versus rivaroxaban for 28 days after hospitalization, with the exact dosing (10, 15 or 20 mg daily) based on an adaptive strategy	Recruitment Status: completed
	ClinicalTrials.gov Identifier: NCT04640181		No results posted
6. ACTIV4c Trial: COVID-19 Post-hospital Thrombosis Prevention Study - Clinical Trial [80]	Ongoing:	Participants will be randomized to either prophylactic anticoagulation (Apixaban 2.5 mg twice daily) or matching placebo for 30 days, and then followed for an additional 60 days after the completion of treatment (total duration of follow-up, 90 days)	Recruiting
	ClinicalTrials.gov Identifier: NCT04650087		
7. Helping Alleviate the Longer-term consequences of COVID-19 (HEAL-COVID): a national platform trial [81]	Ongoing:	This randomized trial will assess the safety and effectiveness of apixaban and atorvastatin in comparison to the standard of care over 12 months after discharge	Recruiting:
	ClinicalTrials.gov Identifier: NCT04801940		Estimated enrollment 2631 patients

Legend: LMWH, low molecular weight heparin; RRR, relative risk reduction; CI, confidence interval.

Finally, to establish the optimal duration and the type of extended thromboprophylaxis the future ongoing studies will help to validate the protocols in different populations: (1) the NCT04508439 study enrolling 130 patients will evaluate prophylactic and full-dose heparin administered in hospital followed by rivaroxaban 10 mg/day or no intervention; (2) the NCT04640181 study is evaluating 150 patients randomized to in hospital enoxaparin or oral rivaroxaban (10 mg/day, 15 mg/day, or 20 mg/day) for 28 days after discharge; (3) the ACTIV-4c (NCT04650087) trial will enroll 4000 patients to assess the safety and effectiveness of apixaban, aspirin or placebo in patients discharged from the hospital; (4) the HEAL-COVID (NCT04801940) randomized trial will enroll patients to assess the safety and effectiveness of apixaban and atorvastatin versus standard of care over 12 months after discharge—Table 1 (Ref. [60, 79, 80, 81]). The evolving new results, together with the data from MICHELLE trial, will probably contribute to new recommendations of the updating guidelines.

## 7. Conclusions and Recommendations

The SARS-CoV-2 infection is associated with increased levels of inflammation and procoagulants, including D-dimer. Those patients with severe symptoms and accentuate inflammatory response had higher rates of thrombosis during hospital stay and must be evaluated for ongoing risk of VTE before discharge. The available data suggest a relatively low incidence of post discharge VTE, less than 2–3%, and currently guidelines advise no routine extended thromboprophylaxis for patients who do not have suspected or confirmed VTE or another indication for anticoagulation. However, the guidelines recommend an individual assessment of the VTE and bleeding risks, to identify those patients who could benefit from ongoing prophylactic anticoagulation. Therefore, there is an unmet need to have more valuable data about the VTE and bleeding outcomes in COVID-19 patients after hospital discharge. The duration and the type of prophylactic anticoagulation are still on debate, but DOACs at low doses compared to prophylactic LMVH was predominantly used in most of the patients.

A summary of the practical recommendations is given below:

∙ All patients with COVID-19 disease should be assessed for ongoing risk of VTE before hospital discharge. A score like IMPROVE-DD is a valuable tool.

∙ Clinical judgment and the balance between VTE and bleeding risks represent the key factors for the use of the extended prophylactic anticoagulation.

∙ Inform patients and their relatives that after COVID-19 the residual risk for VTE events, even if not high, is still present and in selected cases the anticoagulation must continue for a period after hospital discharge.

∙ Inform patients and their relatives about the benefits and risks of different types of extended thromboprophylaxis.

∙ Inform patients and their relatives about the signs and symptoms of thrombosis and urge them to seek urgent medical advice if they suspect a PE or DVT.
